# Molecular Screening and Analysis Reveal Novel Oral Site-Specific Locations for the Cariogenic Pathogen *Scardovia wiggsiae*

**DOI:** 10.3390/dj9060073

**Published:** 2021-06-17

**Authors:** Steven McDaniel, Jaydene McDaniel, Katherine M. Howard, Karl Kingsley

**Affiliations:** 1 Department of Advanced Education in Pediatric Dentistry, School of Dental Medicine, University of Nevada, 1700 W. Charleston, Las Vegas, NV 89106, USA; mcdans1@unlv.nevada.edu (S.M.); mcdanj1@unlv.nevada.edu (J.M.); 2 Department of Biomedical Sciences, School of Dental Medicine, University of Nevada, 1001 Shadow Lane, Las Vegas, NV 89106, USA; katherine.howard@unlv.edu

**Keywords:** *Scardovia wiggsiae*, dental caries, saliva screening

## Abstract

Introduction: *Scardovia wiggsiae* (SW) is a newly identified cariogenic pathogen associated with severe early childhood caries and oral disease. New studies have confirmed the presence of this organism among clinical samples from both pediatric and adult patients. However, the recent discovery of this organism has left researchers with only limited information available regarding the prevalence of this organism—and virtually no information regarding oral site-specific locations. Based upon this lack of information, the overall objective of this study was to perform an oral site-specific analysis of SW prevalence from clinical samples. Methods: Using an approved human subjects protocol, samples (*n* = 60) from an existing saliva and site-specific biorepository were identified and screened for SW presence using quantitative polymerase chain reaction (qPCR). These data were summarized and subsequently analyzed for correlations with demographic (age, sex, race or ethnicity) or clinical (body mass index or BMI, primary/mixed/permanent dentition, orthodontic brackets) variables. Results: These data revealed that average DNA concentrations from all sample sites (saliva, dorsum of tongue, gingival crevicular fluid (GCF), biofilm of upper buccal molar, and biofilm of lower lingual incisor) ranged between 13.74 and 14.69 μg/μL, with an overall average of 14.30 μg/μL ± 1.12 (standard error or SE). qPCR screening revealed a total of *n* = 34/60 or 56.7% of patient samples harboring SW. A total of *n* = 71/170 specific oral sites harbored this organism, with the majority of the SW-positive participant samples harboring SW at more than one oral site, *n* = 22/34 or 64.7%, including non-traditional sites such as GCF and the dorsum of the tongue. Weak correlations were found between specific SW outcomes in GCF and type of dentition (permanent; R = 0.2444), as well as SW outcomes in saliva with age (R = 0.228) and presence of orthodontic brackets (R = 0.2118). Conclusions: This study may be among the first to provide oral site-specific analysis to reveal the prevalence and location of *Scardovia* among clinical patient samples. Moreover, these data also provide some of the first evidence to suggest this organism may be present not only in traditional supragingival tooth-associated biofilm sites, but also in non-traditional oral sites including the dorsum of the tongue and the gingival crevice. Based upon these results, these data may represent a significant advance in our understanding of the potential sites and locations that harbor this organism and may help contribute to our understanding of the prevalence, distribution and potential for the development of oral disease among clinic patients.

## 1. Introduction

*Scardovia wiggsiae* (SW) is a recently identified, Gram-positive cariogenic pathogen strongly associated with poor health outcomes and oral disease [1,2]. More specifically, SW has subsequently been characterized as an anaerobic, Gram-positive bacillus most closely related to *Scardovia inopinata* [3]. Genetic analysis revealed this organism as saccharolytic, with the potential capability for both acetic and lactic acid fermentation pathways [4]. These potential capabilities suggested this organism might therefore be a contributor to the oral biofilm responsible for the development or progression of oral caries [5].

Many studies now confirm the presence and rising levels of this pathogen in cases of severe and early childhood caries [6,7,8]. This organism may represent a unique niche in the oral biofilm, with the ability to metabolize glucose and produce fermentation products lactic, acetic and formics acid with higher fluoride tolerance and lower enolase sensitivity than other cariogenic pathogens, such as *Streptococcus mutans* (SM) [9,10].

The ability for SW and other *Bifidobacteria* to metabolize glucose apart from the fluoride-inhibited enolase and lactic acid-producing pathway through a shunt allowing metabolic activity and the production of acetate to continue has been identified as an important virulence mechanism [10,11,12]. Because the incorporation of fluoride into toothpastes, mouthwashes and topical applications is an important feature of anti-cariogenic oral health strategies, understanding the prevalence of SW and other organisms that may be resistant to fluoride at SM-inhibitory concentrations as well as understanding the oral sites and locations for the aggregation of this organism and methods for altering biofilm composition become increasingly important [13,14,15]. Pilot studies from this group have explored alternative methods for decreasing the cariogenic bacterial burden among children by evaluating the effect of dental sealants, which may exhibit modest and temporary effects on SW—although much remains to be discovered regarding the oral sites and prevalence of this organism [16,17].

Based upon the limited applicability of most cariogenic-inhibiting treatments and therapies to target SW specifically, an understanding of the overall prevalence of this organism has been increasingly important [18,19]. Studies from this group have also evaluated the prevalence and distribution of this organism among pediatric and adult clinical patient samples [20,21]. These studies have confirmed previous work suggesting that orthodontic therapy may be a potential risk factor influencing the growth and development of this organism [22,23]. These have led to additional research from this group to evaluate the populations, including pediatric and adult orthodontic patients, most at risk for harboring this organism [24,25,26].

Most recently, a series of site-specific sampling studies from this group have revealed differential oral locations for SW distribution among patient samples—a major step towards understanding the potential sites for colonization [27,28]. However, due to the limited number of patient samples available in these initial studies, a larger and more comprehensive microbial screening analysis was the overall objective of this current study. By combining a larger study sample and site-specific data with demographic, clinical and orthodontic treatment variables, it is hoped a more complete and thorough understanding of oral location, risk factors and prevalence can be accomplished.

## 2. Materials and Methods

### 2.1. Study Approval

The protocol for this retrospective study was reviewed and approved by the Biomedical institutional review board (IRB) and Office for the Protection of Research Subjects (OPRS) at the University of Nevada, Las Vegas (UNLV) under 1717625-1 titled “Retrospective analysis of microbial prevalence from DNA isolated from saliva samples originally obtained from the University of Nevada, Las Vegas (UNLV) School of Dental Medicine (SDM) pediatric and clinical population”. The original study sample collection was reviewed and approved by the UNLV IRB under OPRS#1305-4466M “The Prevalence of Oral Microbes in Saliva from the UNLV School of Dental Medicine Pediatric and Adult Clinical Population” in 2013.

### 2.2. Sample Collection

The original sample collection was performed in the UNLV SDM clinics. Participation was voluntary and all study participants were required to provide informed consent if over the age of 18 years old or pediatric assent if under the age of 18 years old. Exclusion criteria included any patients (or parents/guardians) that declined to participate and any persons not a patient of record at UNLV-SDM. Inclusion criteria were indication of voluntary participation and provision of informed consent and/or pediatric assent.

In brief, study sample participants were provided sterile saliva collection tubes, which were labeled with randomly generated, non-duplicated numbers to prevent any association with patient identifying information. In addition, paper points were used to collect biofilm samples from the dorsum of the tongue, lingual surface of a mandibular incisor, buccal surface of a maxillary molar, and the gingival crevice of the central maxillary incisor. Basic demographic information for each sample was collected, which included age, sex, race/ethnicity and body mass index (BMI). Clinical information, such as primary/mixed/permanent dentition and the presence of orthodontic brackets, was also collected. All samples were then transferred to a biomedical laboratory for storage at −80 °C, processing and subsequent analysis.

### 2.3. DNA Isolation and Analysis

DNA was isolated from 100 μL of saliva samples and from paper points that were placed into 100 μL of sterile 1X phosphate-buffered saline (PBS) and vortexed to release any bacteria or other microbial constituents. Each sample was then processed using the phenol: chloroform extraction method, as previously described [21,28]. In brief, 300 μL of TriZol was added to each sample and mixed to dissolve microbial membranes and cell walls. A volume of 200 μL of chloroform was added and mixed prior to centrifugation at 12,000× *g* or relative centrifugal force (RCF) for 15 min to separate the proteins and nucleic acids. The aqueous supernatant containing the nucleic acids was removed and transferred to new sterile microcentrifuge tubes and mixed with 100 μL of isopropanol to precipitate the DNA, which was then centrifuged. The pellet was washed with nuclease-free ethanol prior to subsequent centrifugation and resuspension in 100 μL of sterile, nuclease-free water. Each sample was then screened for DNA concentration and purity using a NanoDrop spectrophotometer using absorbance readings at A260 and A280 nm.

### 2.4. qPCR Screening

Samples with sufficient quantity (>10 ng) and quality A260:A280 ratio above 1.70 were then screened for microbial presence using quantitative polymerase chain reaction (qPCR) primers specific for the bacterial positive control (16S rRNA universal primer) and *Scardovia wiggsiae* (SW). In brief, each qPCR screening was performed in duplicate and consisted of a reaction containing 2X ABsolute SYBR green master mix (12.5 μL), forward and reverse primers (1.5 μL each), sample DNA (1.5 μL diluted to 1.0 ng/μL) and distilled nuclease-free water (8.0 μL). Processing was performed using activation at 95 °C for 15 min, followed by 40 cycles involving denaturation (95 °C for 15 s), annealing (primer-specific temperatures below for 30 s) and extension (72 °C for 30 s).

Positive control, bacterial 16S rRNA

Forward 16S rRNA universal primer

5′-ACG CGT CGA CAG AGT TTG ATC CTG GCT-3′; Tm = 76 °C

Reverse 16S rRNA universal primer

5′-GGG ACT ACC AGG GTA TCT AAT-3′; Tm = 62 °C

Annealing temperature = lower Tm (62 °C) − 2 °C = 60 °C.

### 2.5. Scardovia Wiggsiae (SW)

SW Forward primer

5′-GTG GAC TTT ATG AAT AAG C-3′; Tm = 55 °C

SW Reverse primer

5′-CTA CCG TTA AGC AGT AAG-3′; Tm = 56 °C

Annealing temperature = lower Tm (55 °C) − 2 °C= 53 °C.

### 2.6. Statistical Analysis

Demographic variables were summarized and descriptive statistics reported. Any differences between the study sample demographics and the overall clinic population were analyzed using chi square statistics, which is appropriate for categorical, non-parametric data. Parametric data (such as DNA concentration) were summarized and descriptive statistics reported. Any differences between samples were analyzed using two-tailed Students’ *t*-tests, which are appropriate for continuous parametric data analysis. Any associations between demographic or clinical characteristics and the screening outcomes were analyzed using binomial logistic regression analysis.

## 3. Results

A total of *n* = 60 patient samples were identified for inclusion in this study (Table 1). Demographic analysis of the study sample participants revealed that slightly more than one-third were derived from females (*n* = 22/60 or 36.7%), which was significantly different from the overall percentage of females from the clinic population (49.1%), *p* = 0.0164. The percentage of minorities was also much higher within the study sample (*n* = 52/60 or 86.7%) than in the clinic population (65.4%), which was also statistically significant, *p* = 0.00012. Finally, the average age of the study sample was 11.01 years, which was not significantly different from the pediatric clinic patient population of 10.41 years, *p* = 0.441.

To determine whether the DNA isolated from these samples had sufficient quality and quantity for molecular screening using qPCR, analysis was performed using spectrophotometry (Table 2). These data revealed that the average DNA concentration from all sample sites ranged between 13.74 and 14.69 μg/μL, with an overall average of 14.30 μg/μL ± 1.12 (standard error or SE). None of the site-specific averages were significantly different from the overall average of all site combined, *p* = 0.643. DNA purity as measured by the ratio of absorbance at A260 and A280 nm revealed that average purity for all sites exceeded 1.60 (range 1.618 to 1.701), which has been deemed acceptable for molecular screening using high-fidelity methods such as qPCR.

**Figure 1:**
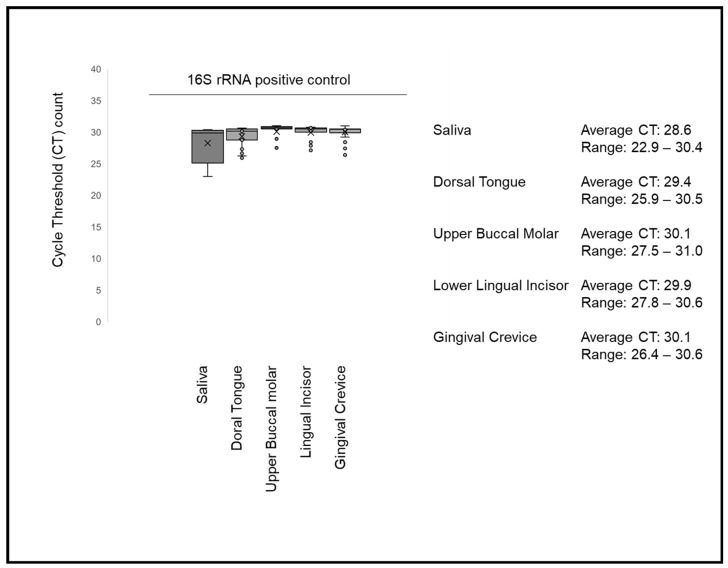
qPCR screening of samples for positive control 16S rRNA. All study samples harbored bacterial DNA with average cycle threshold (CT) counts ranging from 28.6 (saliva and lower lingual incisor), 29.9 (dorsal tongue), to 30.1 (upper buccal molar and gingival crevicular fluid).

Confirmation of bacterial DNA presence from each study sample was accomplished using real-time quantitative polymerase chain reaction (RT-qPCR) using bacterial 16S rRNA universal primers (Figure 1). These data demonstrated that all patient samples and study sites harbored detectable levels of bacterial DNA. More specifically, the cycle threshold counts (CT) for saliva ranged between 22 and 30 (average 28.6), with more narrow ranges observed among the other sites, such as dorsal tongue (range: 25–30; average 29.4), upper buccal molar (range: 27–30, average 30.1), lower lingual incisor (range: 27–31; average 29.9) and gingival crevicular fluid (range: 26–30; average 30.6).

**Figure 2:**
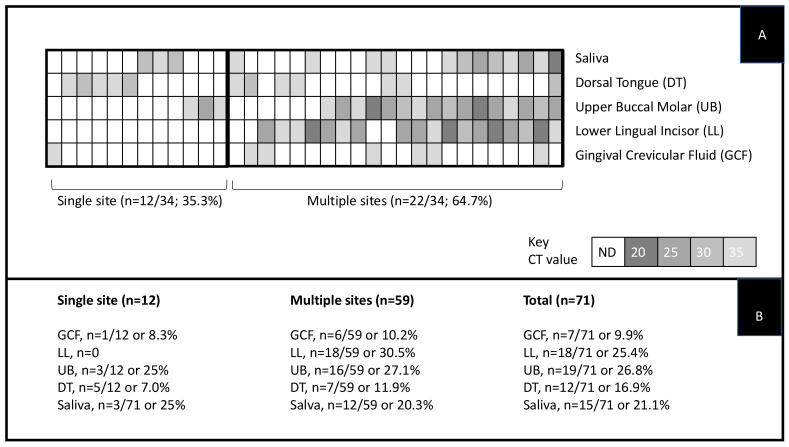
Heat map of study sample molecular screening for *S. wiggsiae*. (**A**) Heat map of the qPCR screening revealed a total of *n* = 34/60 or 56.7% of patient samples harboring SW. (**B**) A total of *n* = 71/170 specific oral sites harbored this organism, with the majority of the SW-positive participant samples harboring SW at more than one oral site, *n* = 22/34 or 64.7%. Note: Key indicates relative DNA abundance through CT values: 20 = greater abundance; 35 = less abundant.

Screening of these samples for SW revealed that more than half of all study sample participants had at least one site harboring this organism, *n* = 34/60 or 56.7% (Figure 2). Analysis of the SW-positive patient samples revealed a total of *n* = 71/170 specific oral sites that harbored this organism, with the majority of the SW-positive participant samples harboring SW at more than one oral site, *n* = 22/34 or 64.7% (Figure 2A). More detailed analysis of these sites revealed that the majority of the single-site SW-positive results came from the dorsal tongue (*n* = 5/12 or 41.7%), with upper buccal molar (*n* = 3/12 or 25%) or saliva (*n* = 3/12 or 25%) comprising the bulk of the remaining sites.

The majority of SW-positive samples harbored SW at more than one oral site, with the most common sites revealed as the lower lingual incisor (*n* = 18/59 or 30.5%) and upper buccal molar (*n* = 16/59 or 27.1%) and saliva (*n* = 12/59 or 20.3%) (Figure 2B). Fewer of the samples with multiple sites harbored SW from the dorsal tongue (*n* = 7/59 or 11.9%) or gingival crevicular fluid (*n* = 6/59 or 10.2%). The combined total of both single- and multi-site data revealed that the most common sites overall were from the biofilm samples derived from the upper buccal molar (*n* = 19/71 or 26.8%) and lower lingual incisor (*n* = 18/71 or 25.4%), with many of these samples also demonstrating SW-positive results from the corresponding saliva (*n* = 15/71 or 21.1%). Overall, fewer SW-positive samples were found among the dorsal tongue (*n* = 12/71 or 16.9%) and gingival crevicular fluid (*n* = 7/71 or 9.9%).

To determine whether there are any significant associations between SW-positive site-specific screening (dependent) outcomes and any potential predictor variables (age, sex, BMI, dentition, brackets), Pearson’s correlation was assessed (Table 3). These data demonstrated that the majority of variables had negligible or very weak associations between the variables analyzed and SW-positive outcomes (between 0.0 and 0.19 or −0.19). However, three weak associations were found between specific SW outcomes and specific variables, which included a correlation between type of dentition (permanent) and SW-positive results with the GCF (R = 0.2444) and both age (R = 0.228) and presence of orthodontic brackets (R = 0.2118) with SW-positive results in saliva.


Key: 
**Strength of Correlation (R)**

**Correlation Coefficient Range**
Negligible/Very Weak0.0–0.19 or −0.19–0.0Weak0.20–0.39 or −0.39–−0.20Moderate0.40–0.59 or −0.40–−0.59Strong0.60–0.79 or −0.79–−0.60Very Strong0.80–1.0 or −1.0–−0.80


## 4. Discussion

The objective of this current study was to facilitate a molecular screening of samples with site-specific oral locations for SW. The results of this study clearly demonstrate that molecular screening and identification of this novel cariogenic pathogen are feasible and effective at determining site-specific locations that may harbor detectable levels, similar to other molecular methods for oral screening and pathogen identification [29,30,31]. In addition, this study may be among the first to describe the presence of this organism in non-traditional oral sites and locations, such as the biofilm on the dorsum of the tongue and GCF, which have not been oral sites or locations associated with the most prevalent cariogenic- and supragingival biofilm-associated organisms, such as *Lactobacillus* spp. and *S. mutans* [32,33,34].

Moreover, this study is among the first to provide an analysis and documentation of the prevalence of single-site versus multi-site SW-positive samples from an oral biorepository, similar to other initiatives that attempted to map microbial distribution and body sites for other types of medically important organisms, such as *Staphylococcus aureus* and *Clostridium difficile* [35]. Although more than half of the sites harboring this organism were traditional supragingival biofilm sites, such as the tooth biofilm of the upper buccal molar and lower lingual incisor, less obvious sites were revealed such as the biofilm from the dorsum of the tongue, which has been demonstrated in other studies to be an important intermediate site for the movement and redistribution of oral microbes [36,37]. In fact, this site has been recently recognized as an increasingly important site that contributes to overall microbial burden and oral ecology through various mechanisms, including salivary redistribution [38,39,40,41].

In addition to these novel insights, this study also provides the first evidence of SW presence from the gingival crevice, an area more traditionally associated with periodontal pathogens and anaerobic Gram-negative microbes [42,43]. Although the majority of GCF-associated SW-positive samples were found among patients with multi-site SW-positive results, at least one single-site positive sample was found to harbor SW only in the GCF—which may suggest that more detailed and specific studies are needed to better understand the potential microbial interactions in the subgingival biofilm and the gingival crevice [44,45,46]. Indeed, there may be undiscovered mechanisms of polymicrobial synergy or other interactions involving SW that more detailed studies in the future may be able to elucidate, thus furthering our knowledge of oral health and disease [47,48].

Despite the significance of these findings, there are some limitations associated with this study which should also be considered when evaluating this evidence. For example, at least one significant difference was found between the ethnic and racial composition of the study sample and the overall clinic demographics, which may suggest additional methods and protocols may be needed in future studies to reduce the potential for sampling and enrollment bias [49,50]. In addition, there are limitations associated with the inferences that can be made from this study due to the limitations of this pilot study sample size, a common feature among many clinical and epidemiologic studies that attempt to produce relevant clinical data without significant external funding sources [51,52].

## 5. Conclusions

This study may be among the first to provide oral site-specific analysis to reveal the prevalence and location of *Scardovia* among clinical patient samples. Moreover, these data also provide some of the first evidence to suggest this organism may be present not only in traditional supragingival tooth-associated biofilm sites, but also in non-traditional oral sites including the dorsum of the tongue and the gingival crevice. Based upon these results, these data may represent a significant advance in our understanding of the potential sites and locations that harbor this organism and may help contribute to our understanding of the prevalence, distribution and potential for the development of oral disease among clinic patients.

## Figures and Tables

**Table 1:** Demographic analysis of study sample.

	Study Sample	Clinic Population	Statistical Analysis
**Sex**			
Females	*n* = 22/60 (36.7%)	49.1%	Χ^2^ = 5.762, d.f. = 1
Males	*n* = 38/60 (63.3%)	50.9%	*p* = 0.0164
**Race/Ethnicity**			
White	*n* = 8/60 (13.3%)	34.6%	Χ^2^ = 21.275, d.f. = 1
Minority	*n* = 52/60 (86.7%)	65.4%	*p* = 0.00012
Hispanic	*n* = 40/60 (66.7%)	58.6%	
Black	*n* = 8/60 (13.3%)	10.2%	
Asian	*n* = 40/60 (6.7%)	6.6%	
**Age**			
Average age	11.01 years	10.41 years	*p* = 0.441
Range	5–17 years	0–17 years	

**Table 2:** Analysis of DNA isolated from study samples.

	DNA Concentration (Average ± Standard Error or SE)	DNA Purity A260:A280	Statistical Analysis
Gingival Crevice	14.69 μg/μL ± 1.24 SE	1.701 ± 0.21	*p* = 0.722
Upper Buccal Molar	14.34 μg/μL ± 1.17 SE	1.691 ± 0.24	*p* = 0.887
Lower Lingual Incisor	14.40 μg/μL ± 1.16 SE	1.672 ± 0.27	*p* = 0.772
Dorsal Tongue	14.34 μg/μL ± 1.05 SE	1.637 ± 0.31	*p* = 0.911
Saliva	13.74 μg/μL ± 1.01 SE	1.618 ± 0.28	*p* = 0.643
**Average**	14.30 μg/μL ± 1.12 SE	1.664 ± 0.26	

**Table 3:** Correlation of variables to SW-positive site-specific screening results.

	Age	Sex	BMI	Dentition	Brackets
Gingival Crevice	R = −0.1945	R = 0.0611	R = 0.0950	**R = 0.2444**	R = −0.1437
Upper Buccal Molar	R = −0.0810	R = −0.0024	R = −0.0598	R = −0.0010	R = −0.0848
Lower Lingual Incisor	R = −0.0246	R = −0.1057	R = −0.0278	R = −0.1046	R = −0.0509
Dorsal Tongue	R = −0.1604	R = 0.1211	R = 0.0833	R = 0.1550	R = −0.0667
Saliva	**R = 0.2228**	R = 0.0399	R = −0.1701	R = −0.1139	**R = 0.2118**

## Data Availability

The data presented in this study are available on request from the corresponding author. The data are not publicly available due to the study protocol data protection parameters requested by the IRB and OPRS for the initial study approval.
